# Exploring the Italian Experience with Long-Acting Buprenorphine Formulations (LAIB) for the Treatment of Opioid Use Disorder: A Series of Narrative Interviews

**DOI:** 10.3390/ijerph23030336

**Published:** 2026-03-07

**Authors:** Vincenza Ariano, Anna Francesca Costanzo, Gemma Ferrante, Rossella Garofano, Vincenzo Lamartora, Sergio Manfré, Deborah Nordici, Lorenzo Somaini

**Affiliations:** 1 Department of Pathological Addictions, Local Health Authority (ASL) Taranto, 74121 Taranto, Italy; 2 Azienda Socio-Sanitaria Territoriale Fatebenefratelli-Sacco (ASST Fatebenefratelli-Sacco), Addiction Services (Ser.D) Piazzale Accursio 7, 20149 Milano, Italy; 3 Department of Pathological Addictions, Addiction Services (Ser.D) Pozzuoli, 80078 Pozzuoli, Italy; 4 Department of Mental Health and Addiction, Addiction Services (Ser.D) Avellino, 83100 Avellino, Italy; 5 Addiction Service, Ser.T Monza, 20900 Monza, Italy; 6 Departmental Addictions Area—Asugi, 34128 Trieste, Italy; 7 Department of Addiction Treatment Centres, Local Health Authorities of Biella, Novara, Vercelli and Verbano-Cusio-Ossola (ASL BI, NO, VC, VCO), 13900 Biella, Italy

**Keywords:** long-acting buprenorphine, opioid agonist therapy, narrative medicine, patient experience, LAIB, patient engagement

## Abstract

**Highlights:**

**Public health relevance—How does this work relate to a public health issue?**

- Opioid Use Disorder is a chronic public health condition requiring long-term, sustainable treatment strategies that extend beyond symptom control.
- Long-acting buprenorphine (LAIB) formulations address structural and psychosocial barriers associated with daily opioid agonist therapy.

**Public health significance—Why is this work of significance to public health?**

- The present analysis provides the first narrative-based evidence from Italy on patient experiences with long-acting injectable buprenorphine.
- Findings show that treatment effectiveness in OUD includes autonomy, stigma reduction, and psychosocial recovery, not only pharmacological stabilization.

**Public health implications—What are the key implications or messages for practitioners, policy makers and/or researchers in public health?**

- Incorporating patient narratives into addiction care can inform more personalized, recovery-oriented, and acceptable treatment pathways.
- LAIB may support broader public health goals by improving adherence, social reintegration, and long-term treatment sustainability.

**Abstract:**

Long-Acting Buprenorphine Formulations (LAIB) have emerged as an alternative pharmacological approach for opioid use disorder, offering potential benefits extending beyond clinical stabilisation. Narrative medicine provides a unique approach to understand patients’ perspectives and experiences with sublingual buprenorphine and LAIB dispensed to fourteen patients across different Italian Addiction Services, examining how they impact the emotional, social, and motivational dimensions of recovery. Narratives were analysed by thematic content across eight domains: dependence on daily treatment regimen, emotional impact, self-perception, determination to change, quality of life, craving and withdrawal symptoms, treatment adherence, social burden, and therapeutic relationship. Statements were categorised by valence; experiential patterns were qualitatively analysed. Sublingual buprenorphine, although effective, was associated with reduced autonomy, symptom control, and difficulties in balancing treatment, work and life. These aspects were correlated with worse adherence. The stigma and burden of daily intake can reduce motivation and hinder identity reconstruction. In this setting, transitioning to LAIB resulted in improved self-autonomy, emotional balance, symptom control, self-esteem, and reduced daily and psychological burden, craving and stigma, facilitating social reintegration, and strengthening the therapeutic relationship. The results emphasise the importance of including both experiential and narrative elements in clinical care, as this helps create more tailored, recovery-focused treatment pathways.

## 1. Introduction

Opioid use disorder (OUD) remains a critical global public health challenge. According to the World Health Organisation [1] and the United Nations Office on Drugs and Crime [2], approximately 61 million people worldwide used opioids in 2022, representing about 1.2% of the global population aged 15–64 years, while an estimated 36 million individuals met diagnostic criteria for opioid dependence. The global burden of disease attributable to opioid use has increased by more than 70% since 1990, reflecting both the rising availability of synthetic opioids and the continued prevalence of heroin use. In Europe, data from the European Monitoring Drug Agency [3] indicate that opioids remain the primary driver of drug-related mortality, accounting for approximately 74% of fatal overdoses. Moreover, 860,000 people in the European Union engage in high-risk opioid use, corresponding to roughly 0.4% of the adult population [3]. These figures underscore both the chronicity of the opioid use disorder and the urgent need for effective, accessible, and sustainable treatment approaches.

In Italy, national surveillance data confirm a stable yet substantial burden of opioid dependence. According to the Dipartimento delle Politiche contro la Droga e le altre Dipendenze, public Addiction Services (Ser.D) treated 129,259 individuals with substance use disorders in 2022, of whom about 63% reported opioids as their primary substance of abuse [4]. The majority were men aged 35–54 years, reflecting a population with long-standing opioid dependence rather than newly emerging users. Among new entrants into treatment, 34.6% reported opioid use as their main problem substance, compared to 67.4% among those already in or returning to treatment [4]. These data indicate that, while the absolute prevalence of opioid use in the general Italian population remains lower than in some other regions, opioid dependence continues to represent a major component of Italy’s treated addiction population and a significant public health issue.

OUD is a chronic relapsing condition characterised by compulsive opioid use, tolerance, and withdrawal, resulting in profound biological, psychological, and social consequences. OUD is characterised by a persistent pattern of compulsive opioid use leading to clinically significant impairment, often accompanied by tolerance, withdrawal, craving and relapse. The disorder significantly impacts physical, psychological, and social functioning. Opioid agonist treatment (OAT), when integrated with psychosocial support, is recognised as the evidence-based standard intervention for OUD. Research indicates that OAT substantially reduces all-cause and overdose mortality, while promoting patient stabilisation, reducing cravings and opioid use, preventing overdose, supporting psychosocial rehabilitation, and enhancing recovery outcomes [5,6].

Among the different OATs, methadone and sublingual buprenorphine are considered the standard of care for the treatment of OUD. They are characterised by different pharmacodynamic and pharmacokinetic profiles. Buprenorphine is a partial μ-opioid receptor agonist and κ-opioid receptor antagonist, characterised by high receptor affinity, lower agonist potency, and a ceiling effect on respiratory depression [7,8]. Its partial agonism ensures effective suppression of withdrawal and craving with a lower risk of respiratory depression compared to methadone. However, sublingual buprenorphine requires daily or near-daily administration, which can increase logistical burden, limit patient autonomy, perpetuate stigma, and reduce treatment adherence [9]. This is particularly relevant in the Italian context, which differs substantially from other European countries and the United States. The 570 public Ser.D, operating across 610 sites nationwide, provide free-of-charge care and are the only legally authorized centers for diagnosing OUD and defining treatment plans [10]. Within this system, supervised administration of sublingual buprenorphine remains common, particularly in early treatment phases [11], and may influence patients’ subjective treatment experience. Furthermore, for sublingual buprenorphine, the risk of misuse and diversion is well documented [12,13,14]. To address some concerns about sublingual buprenorphine, Long-Acting Injectable Buprenorphine (LAIB) formulations, extended-release depot preparations administered subcutaneously by healthcare professionals on a weekly or monthly basis, have been developed [15,16]. Different clinical studies have shown that LAIB is non-inferior to sublingual buprenorphine in key clinical outcomes, including treatment retention and reduction in non-prescribed opioid use [17,18,19]. In addition, owing to supervised administration and sustained therapeutic plasma concentrations, it reduces the risk of medication diversion while limiting opportunities for misuse [17,18,19]. Furthermore, real-world and qualitative studies previously reported potential benefits in treatment adherence, perceived treatment burden, emotional stability, stigma reduction, and quality of life (QoL) compared with daily self-administered formulations [20,21,22,23].

A comprehensive understanding of OUD also requires acknowledging the multidimensional staging framework that characterises its treatment. Evidence—based models describe five interrelated phases: induction, stabilisation, maintenance, tapering/disengagement (when clinically appropriate and aligned with the patient’s recovery trajectory), and relapse, each supported by distinct neurobiological and clinical outcomes. Notably, tapering is not considered a routine or obligatory component of treatment, as for many individuals, long-term or indefinite maintenance represents the recommended and most effective therapeutic strategy.

Despite a growing body of evidence from different countries [20,21,23,24] on the efficacy of LAIB, to date, no research has examined the experiences of Italian patients receiving such formulations. Given the distinctive features of the Italian addiction care system—characterised by publicly funded, multidisciplinary services integrated into Local Health Systems—understanding patients’ perspectives within this setting is crucial for guiding implementation and evaluating real-world effectiveness. The increasing international emphasis on patient-centred and holistic approaches further reinforces the importance of integrating subjective experience into evaluations of therapeutic efficacy [25].

To this end, we adopted the methodological framework of Narrative Medicine, a qualitative approach that foregrounds the patient’s lived experience as an essential component of clinical evaluation [26]. Narrative Medicine explores how individuals understand their illness, treatment, and recovery, providing insight into the meanings and values that shape therapeutic change. In the context of long-acting treatment for OUD, this perspective allows for a deeper understanding of how pharmacological stabilisation translates into enhanced autonomy, identity reconstruction, and reintegration into social life.

This preliminary research analyses narratives from fourteen patients with OUD who received LAIB treatment for several months. Using a shared semi-structured interview framework, the study examines, by means of Narrative Medicine, how patients describe their treatment trajectories—from their initial encounter with opioids to their decision to initiate treatment, the process of transitioning to LAIB formulations, and the perceived changes in daily functioning and well-being.

## 2. Materials and Methods

### 2.1. Study Design

This qualitative study was conducted using a Narrative Medicine approach to explore patients’ lived experiences during the transition from sublingual buprenorphine to LAIB. The study was performed by using a semi-structured interview format, combining inductive thematic analysis with a descriptive, semi-quantitative assessment of positive and negative experiential statements across thematic domains.

### 2.2. Participants

Fourteen patients diagnosed with OUD and treated by different Italian Addiction Services (Ser.D) were included. Patients were recruited from Ser.D located in different regions (Friuli Venezia Giulia, Lombardia, Campania, and Apulia) to ensure a fair representation of Northern and Southern Italy. A purposive sampling strategy was adopted to capture heterogeneity in age, gender, and treatment history. All participants had previous experience with sublingual buprenorphine and had been stabilised on LAIB for at least three months at the time of the interview. The LAIB was administered subcutaneously by healthcare professionals on a weekly or monthly basis, as per routine clinical practice. Recruitment continued until thematic saturation was achieved, defined as the point at which successive interviews did not generate substantially new codes or conceptual categories. Saturation was observed after approximately twelve interviews; two additional interviews were conducted to confirm redundancy and thematic stability. Informed consent was obtained from all subjects involved, and confidentiality and anonymity were ensured throughout data collection, transcription, analysis, and reporting.

### 2.3. Data Collection

Interviews were conducted between September and November 2025 in Italian by trained clinicians experienced in qualitative interviewing. All participants were fluent in Italian; although one participant was of non-Italian origin, sufficient language proficiency was confirmed before inclusion. During the interviews, language and phrasing were adapted to each participant’s educational level to ensure clarity, accurate understanding of the questions, and meaningful narrative expression. A standard interview guide was used to conduct the semi-structured interviews. These focused on participants’ experiences with sublingual buprenorphine, transitioning to LAIB, and perceived changes after starting the new treatment. The first section of the interview focused on the experience with sublingual buprenorphine, including perceived benefits, limitations, and the impact on daily life. The second section reviewed the transition to LAIB, focusing on expectations, concerns, and early adaptation. The last section assessed how LAIB treatment impacted emotional health, social and work performance, and motivation to recover. All interviews were conducted in person or by secure video call, recorded with consent, and transcribed verbatim for analysis.

### 2.4. Narrative Analysis and Coding Procedure

The interviews were examined using thematic content analysis based on Narrative Medicine principles. Each statement was treated as a unit of meaning and evaluated for positive or negative valence regarding specific treatments (sublingual buprenorphine or LAIB), then categorised into themes reflecting main aspects of patients’ experiences. Two independent raters coded the data separately.

### 2.5. Thematic Areas

#### 2.5.1. Time and Personal Freedom

This category included statements referring to the management of daily time, routine, and the perceived freedom resulting from reduced treatment frequency. Examples: “I no longer have to think about therapy every day” (positive) or “I had to organise my days around the medication” (negative).

#### 2.5.2. Economic and Practical Aspects

Statements describing direct or indirect costs, as well as practical difficulties in attending the service, were grouped in this area. Examples: “I no longer lose workdays to go to the clinic” (positive) or “Every day I had to travel and spend money to get there” (negative).

#### 2.5.3. Perception of Dependence and Autonomy

This theme encompassed reflections on the sense of dependence on the medication or on the healthcare system and the recovery of personal autonomy. Examples: “I feel less tied to the medication” (positive) or “I depended on schedules and staff” (negative).

#### 2.5.4. Emotional Experience

To this category were assigned statements referring to clearly identifiable emotions such as fear, confidence, anxiety, anger, or serenity. Examples: “At the beginning I was afraid of the injection” (negative) or “After the first administration I felt relieved” (positive).

#### 2.5.5. Impact on Daily Life

This domain included statements describing how treatment influenced family life, social relations, and the ability to work or engage in everyday activities. Examples: “Since starting this therapy, I have gone back to work” (positive) or “With the previous therapy, I was always tired” (negative).

#### 2.5.6. Barriers and Adherence Difficulties

Statements referring to practical or psychological obstacles in following the therapy were grouped in this theme. Examples: “I had to remember to take it every day” (negative) or “Before starting, I was afraid of the injection” (negative).

#### 2.5.7. Craving and Control of Desire

This area included statements concerning craving intensity, its reduction, or the sense of stability achieved with treatment. Examples: “I no longer think about drugs as before” (positive) or “With the oral therapy, I still had cravings” (negative).

#### 2.5.8. Motivation and Determination to Change

This category encompassed statements expressing personal motivation, determination, or readiness to change and progress in recovery. Examples: “For the first time, I believe I can make it” (positive) or “I was not yet ready to change” (negative).

#### 2.5.9. Craving Combined with Adherence Difficulties

Statements that simultaneously expressed craving and difficulty in maintaining adherence were included here. Examples: “When I skipped a dose, the desire came back” (negative) or “I was afraid that the treatment would not last long enough” (negative).

#### 2.5.10. Overall Impact on Life

This domain included statements reflecting the overall perception of how treatment affected daily life, time management, work, and general well-being. Examples: “Now I can plan my days” (positive) or “Before, therapy influenced everything” (negative).

## 3. Results

The analysis of fourteen narrative interviews revealed complex and heterogeneous life trajectories preceding OUD, followed by distinct experiential patterns associated with sublingual buprenorphine and, subsequently, with LAIB. Narratives addressed not only the clinical effectiveness of treatments, but also their psychological, social, and existential impact.

### 3.1. Life Background and Early Context

Participants recounted varied backgrounds influencing later substance use: some had stable upbringings with supportive families and activities, while others experienced early adversity such as parental conflict and emotional neglect. These experiences often coincided with school disengagement, emotional instability, or premature entry into the workforce.

Despite notable heterogeneity among patients, a common pattern involved the progressive build-up of emotional tension—often associated with familial disruption or identity challenges—which patients subsequently identified as risk factors for initiating drug use. Adolescence was consistently highlighted as a critical stage wherein social integration, the pursuit of emotional relief, and impulsive tendencies converged, thereby fostering an environment conducive to substance initiation (see Table 1).

### 3.2. Initiation and Escalation of Opioid Use

Substance use disorder typically began in adolescence with alcohol, cannabis, or stimulants, before progressing to opioids in young adulthood. Certain patients began using heroin in settings involving close peer associations or nightlife venues where there was easy access to drugs. Some described using heroin for its calming or numbing effects to relieve anxiety, trauma, or family distress (Table 2).

Escalation from experimentation to daily opioid use often occurred rapidly once withdrawal symptoms emerged. Several patients shifted from smoking to injecting heroin, while others combined opioids with stimulants or sedatives. The consequences were consistently serious, leading to physical decline, family disputes, broken relationships, social isolation, unemployment, financial problems, and engagement in dangerous or illegal behaviour (see Table 3). These cumulative harms prompted all participants to seek care at Ser.D services.

### 3.3. Experience with Standard-of-Care Treatment

**Figure 1:**
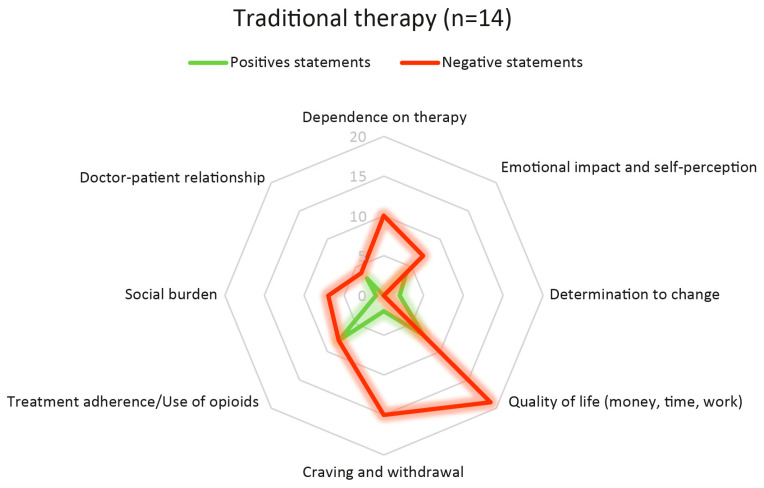
Spider plot illustrating positive (green line) and negative (red line) statements regarding traditional therapy, classified across the eight thematic domains (dependence on therapy; emotional impact and self-perception; determination to change; QoL—money, time, work; craving and withdrawal; treatment adherence/use of opioids; social burden; doctor–patient relationship). The distances from the centre represent the number of coded statements. The number of positive and negative statements for each category is listed in the table.

Analysis of eight themes showed mostly negative experiences with sublingual buprenorphine (Figure 1 and Table 4). Patients often found daily treatment burdensome and restrictive, impacting their autonomy, emotional health, quality of life, symptom stability, and social functioning.

The domain concerning dependence on daily treatment demands was characterised by a uniformly negative perception, with 10 negative statements and no positive statements. Patients consistently described sublingual buprenorphine as creating a sense of dependence, mainly because of strict dosing schedules and the need for daily visits to Ser.D services. They reported feeling tied to treatment routines, experiencing little flexibility and less control over their own time.

Regarding emotional impact and self-perception, participants generated 7 negative statements and 4 positive ones. While some noted instances of partial stabilisation, the prevailing emphasis within their narratives was on feelings of frustration, shame, and a continual perception of being characterised by addiction. Daily clinic visits often intensified perceived stigma and undermined self-esteem. While sublingual buprenorphine helped several patients manage their symptoms, it was not enough to aid in rebuilding their sense of self or promote emotional healing.

The domain of determination to change included only 2 positive and no negative statements. Motivation was rarely attributed to the treatment itself and was instead linked to personal circumstances, such as family distress, financial pressure, or physical deterioration. Sublingual buprenorphine was seen as effective for managing acute symptoms, but its ability to support ongoing motivation or engagement in recovery was considered limited.

Quality of life was one of the most negatively connoted domains, with 19 negative and only 7 positive statements. Patients consistently reported that daily intake interfered with employment, increased travel-related expenses, consumed significant time, and generated an additional financial burden. Even when some benefits were acknowledged, sublingual treatment was widely perceived as constraining everyday functioning and limiting opportunities for social and occupational reintegration.

Similarly, narratives concerning craving and withdrawal symptoms were largely negative, with 15 negative and 2 positive statements, reflecting persistent instability in symptom control. Patients often reported experiencing fluctuations in their symptoms throughout the day, which led to increased anxiety, concerns regarding withdrawal, and a persistent sense of physiological vulnerability even during treatment.

Participants expressed mixed feelings about treatment adherence and opioid use, offering 8 positive statements and 8 negative ones. Some participants reported improved adherence and reduced opioid use; however, others described inconsistent dosing, episodes of relapse, or efforts to conceal ongoing use, indicating that standard therapy did not uniformly support behavioural stabilisation.

Social burden emerged as another strongly negative domain, with 7 negative and only 1 positive statement. Patients highlighted the stigma linked to visiting Ser.D services, the challenges of concealing their treatment from employers or colleagues, and the emotional burden of being openly recognised as patients. Daily intake was often perceived as reinforcing social marginalisation rather than supporting reintegration.

Finally, perceptions of the doctor–patient relationship were mixed, with 4 negative and 3 positive statements. While some participants expressed appreciation for their clinicians and reported feeling supported, others perceived their interactions as hurried, procedural, or limited by time constraints. Among those who felt judged or feared negative consequences—such as reductions in take-home medication—difficulties in openly disclosing ongoing opioid use were commonly reported.

### 3.4. Experience with Long-Acting Subcutaneous Buprenorphine (LAIB)

**Figure 2:**
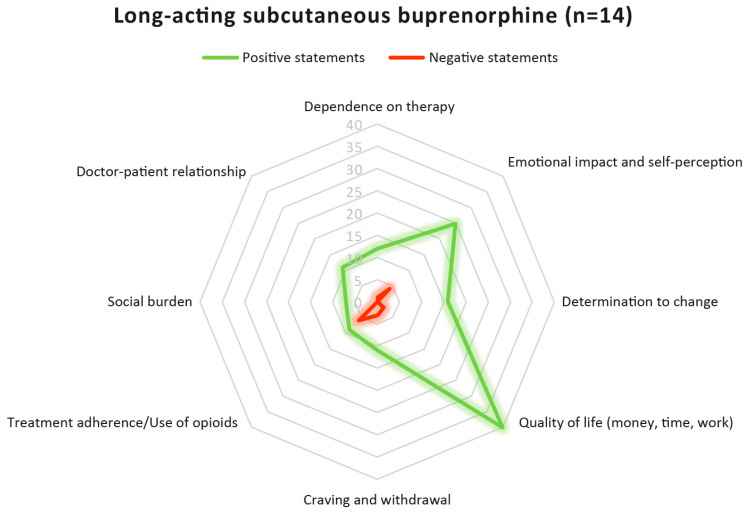
Spider plot illustrating the distribution of positive (green line) and negative (red line) statements related to long-acting subcutaneous buprenorphine, coded across the same eight thematic domains (dependence on therapy; emotional impact and self-perception; determination to change; QoL—money, time, work; craving and withdrawal; treatment adherence/use of opioids; social burden; doctor–patient relationship). The distance from the centre represents the number of statements coded within each domain. The number of positive and negative statements for each category is listed in the table.

Long-acting subcutaneous buprenorphine was associated with a marked shift toward positive experiences across all thematic domains (Figure 2 and Table 5). Compared with sublingual buprenorphine, narratives consistently reflected improvements in autonomy, emotional well-being, QoL, symptom stability, social functioning, and the perceived quality of the therapeutic relationship.

The domain related to dependence on daily treatment requirements was marked by an overwhelmingly positive perception of increased autonomy, as evidenced by 12 favourable statements and only 1 negative comment. Patients emphasised that monthly injections eliminated the need for daily attendance, substantially reduced logistical constraints, and enabled greater flexibility in planning everyday activities without fear of withdrawal or ongoing treatment management. The single negative remark referred to initial uncertainty during the early phase of treatment rather than to sustained dissatisfaction.

Emotional impact and self-perception showed one of the most pronounced positive shifts, with 25 positive and 4 negative statements. Patients experienced improved emotional stability, confidence, and self-identity. Less frequent clinic visits led to reduced stigma and higher self-esteem.

In the domain of determination to change, all statements were positive (16 positive, 0 negative). LAIB was perceived as reinforcing motivation by providing continuity, stability, and a tangible sense of improvement. Many participants shifted their focus from managing symptoms in the short term to pursuing long-term goals.

Quality of life Emerged as the most positively connoted domain, with 40 positive and only 2 negative statements. Patients reported enhanced occupational stability, greater flexibility, reduced time loss, improved productivity, and the ability to travel or engage in routine activities without restrictions. In this domain, the contrast with sublingual buprenorphine was particularly pronounced.

Narratives concerning craving and withdrawal symptoms also reflected substantial improvement, with 11 positive and 3 negative statements. Patients described a sense of well-being maintained throughout the month, less intrusive craving, and reduced anxiety related to physiological fluctuations. Occasional negative remarks were mainly related to the early adjustment period following treatment initiation.

Experiences related to treatment adherence and opioid use included 9 positive and 6 negative statements. The simplified dosing regimen was frequently associated with improved adherence and a reduction in behavioural cues for relapse. Negative statements mainly indicated early instability after switching from sublingual buprenorphine or stemmed from unrelated situations.

Social burden was uniformly positive, with 7 positive and no negative statements. Monthly administration reduced treatment visibility, eliminated the need for daily clinic attendance, and facilitated social integration. Patients frequently reported increased self-esteem and a reduction in perceived stigma.

Finally, perceptions of the doctor–patient relationship were homogeneously positive (11 positive, 0 negative statements). Trust in clinicians was described as central to accepting the treatment change, and reduced visit frequency did not weaken the therapeutic alliance. On the contrary, relationships were perceived as more focused on recovery goals and personal challenges.

### 3.5. Cross-Phase Comparative Synthesis

The cross-phase analysis demonstrates a distinct and consistent difference between experiences reported during sublingual treatment and those observed following the transition to LAIB.

During sublingual buprenorphine treatment, narratives were dominated by negative statements in domains related to autonomy and daily functioning, emotional experience, QoL, craving stability, and social burden. Patients exclusively expressed negative views about daily treatment dependence (10 negative, 0 positive), and mostly negative feelings about emotional impact and self-perception (7 negative, 4 positive), quality of life (19 negative, 7 positive), craving and withdrawal symptoms (15 negative, 2 positive), and social burden (7 negative, 1 positive). Treatment adherence and opioid use experiences were split evenly between positive and negative (8 each), while statements about determination to change (2 positive) and doctor–patient relationships (3 positive, 4 negative) were fewer and more varied.

Following the transition to LAIB, narratives shifted markedly toward positive experiential valence across all domains. Dependence on daily treatment demands was predominantly positive (12 positive vs. 1 negative statement), reflecting increased autonomy and flexibility. Emotional impact and self-perception showed a strong positive rebalancing (25 positive vs. 4 negative statements), as did determination to change (16 positive, 0 negative statements). Quality of life exhibited the most pronounced contrast, with overwhelmingly positive accounts under LAIB (40 positive vs. 2 negative statements). Improvements were also evident in craving and withdrawal stability (11 positive vs. 3 negative statements) and in social burden, which was exclusively positive (7 positive, 0 negative statements). Treatment adherence and opioid use remained more heterogeneous but shifted toward a positive balance (9 positive vs. 6 negative statements). Perceptions of the doctor–patient relationship were uniformly positive after transition (11 positive, 0 negative statements).

Overall, this comparative analysis demonstrates that while sublingual buprenorphine treatment is primarily effective for acute symptom stabilisation, it is often linked with functional, emotional, and social limitations. By comparison, LAIB is associated with comprehensive improvements in patients’ lived experiences, including greater autonomy, enhanced emotional regulation, improved quality of life, increased social integration, and a more positively regarded therapeutic relationship.

## 4. Discussion

This study examined fourteen patients with opioid use disorder who switched from sublingual buprenorphine to LAIB, using narrative medicine and thematic analysis. The results demonstrate that treatment formulation significantly influences not only clinical stabilisation, but also patients’ emotional experiences, perceived autonomy, and capacity to incorporate treatment into their daily lives. In addition to the changes noted following the transition, the narratives underscored several factors that promoted patient engagement with LAIB. Notably, clinicians played a pivotal role by recommending the treatment, offering reassurance, and fostering trust. Furthermore, patients anticipated a reduced treatment burden, enhanced autonomy, and progress aligned with recovery objectives (refer to Supplementary Table S1).

Life histories revealed heterogeneous backgrounds, in which early protective factors coexisted with vulnerabilities such as emotional neglect, family instability, school disengagement, and exposure to peer environments that normalised substance use. Despite these differences, the consequences of OUD were consistently severe, including physical deterioration, financial and occupational instability, relational breakdown, progressive isolation, and loss of personal identity. These accounts show that OAT is crucial not just for controlling symptoms, but also for supporting psychosocial recovery.

Experiences with sublingual buprenorphine demonstrated ambivalence among users. Its efficacy in alleviating withdrawal symptoms and achieving fundamental stabilisation was recognised; however, the requirement for daily or near-daily administration was commonly regarded as inconvenient. The predominance of negative statements in domains related to autonomy, QoL, craving stability, and social burden reflects the impact of daily intake on self-perception and everyday functioning. Patients commonly experience emotional fatigue, encounter stigma linked to their visibility at the Ser.D, and fitting treatment into their professional and social routines. Even when benefits were recognised, experiences often remained fragile and accompanied by vulnerability or fear of judgment. Moreover, variability in access to psychological support across treatment histories further influenced feelings of stability, vulnerability, and engagement with care (see Supplementary Table S2).

In contrast, the transition to LAIB was associated with a marked shift toward positive experiential valence across all thematic domains. Monthly administration substantially reduced the practical and psychological burden of treatment, allowing patients to regain control over time, mobility, and privacy. This change was closely linked to improvements in perceived autonomy, emotional balance, and self-esteem. Craving and withdrawal symptoms were perceived as more stable over time, reducing anxiety related to bodily fluctuations and strengthening everyday functioning. Importantly, reduced visit frequency was not perceived as weakening the doctor–patient relationship; rather, therapeutic alliances were often described as more collaborative and more focused on broader recovery goals than on procedural management. These findings are in line with previous qualitative and mixed-methods studies on LAIB, which describe greater perceived normality, flexibility, and QoL [19,20,21,22,23].

From a narrative standpoint, minimising the frequency of daily reminders and treatment-related rules seemed to influence the way patients described their personal experiences and perspectives. As daily dosing became less central, patients’ narratives shifted from symptom management to emphasising stability, increased personal agency, and future planning. While this study does not permit causal conclusions, the consistent positive experiences observed across various domains indicate that LAIB formulations may facilitate recovery not only via their pharmacological properties but also by influencing the integration of treatment into daily life.

Patients’ accounts offer valuable perspectives on the circumstances where LAIB can be especially significant. In this sample, the motivation to transition was frequently associated with emerging or unstable life equilibria, including employment, family responsibilities, or personal initiatives, which individuals sought to safeguard from the limitations imposed by routine treatment. These results indicate that the patients’ perceptions of treatment burden, along with their desire to preserve specific aspects of daily life, are critical considerations for shared decision-making. This interpretation aligns with patient-centred approaches and is supported by recent qualitative research conducted among healthcare professionals [27].

Several clinical implications emerge from this study. First, treatment burden itself appears to be a central therapeutic dimension: the possibility of leading a life not structured around daily medication may enhance satisfaction, engagement, and emotional well-being. Second, the strong links between formulation, stigma, and self-perception highlight the importance of addressing emotional and identity-related dimensions alongside symptom control. Integrating narrative-based or psychologically informed approaches may further amplify the benefits of long-acting therapy. Third, the perceived improvement in the therapeutic alliance after the transition suggests that involving patients in shared decision-making, building trust, and tailoring treatment plans to individual life circumstances may play a key role in sustaining engagement and continuity of care. The results suggest that LAIB may be suitable not just for patients with poor adherence, but also for those who feel that daily treatment routines interfere with their work, relationships, or personal goals [19,23].

Beyond the individual clinical encounter, the implementation of long-acting buprenorphine can be optimised through organisational models that support continuity and integration across various care settings. Collaborative strategies that engage specialist addiction services, primary care providers, and social services have been linked to enhanced access to, and retention in, opioid use disorder treatment [28,29]. Studies conducted in emergency departments and low-threshold services indicate that engagement is better sustained when patients are guided through clearly defined referral processes and supported transitions from one level of care to another [30,31]. In parallel, digital and telematic tools—including smartphone-based interventions and digital contingency management—have shown promise in supporting retention and reducing opioid use when integrated with pharmacological treatment, suggesting a potential role in maintaining follow-up during extended dosing intervals [32].

Structured organisational frameworks for LAIB have been implemented in countries like Scotland and Australia. These models prioritise patient-centred eligibility, shared decision-making, interprofessional collaboration, flexible service delivery, and provide clear operational guidance [33,34].

However, in Italy, there are still only a few nationally recognised clinical and organisational frameworks that specifically deal with LAIB. Although buprenorphine is well established within Ser.D services, current national guidelines predominantly address sublingual formulations and lack comprehensive, structured recommendations regarding eligibility criteria, service organisation, or integrated care pathways for long-acting injectable preparations [35]. Consequently, current implementation appears to rely largely on local initiatives rather than standardised system-wide models. While the present findings may contribute to informing future discussions on service organisation and guideline development within the Italian context, some limitations should be considered when interpreting the results. The sample size was limited and not designed to be representative of the broader population of individuals with OUD. Retrospective narratives were collected following the transition to LAIB, which may have introduced a positive recall bias. As a qualitative study, this design does not permit conclusions regarding comparative effectiveness or long-term outcomes. Furthermore, the organizational context of OAT in Italy, where sublingual buprenorphine is frequently administered under supervised conditions, represents a confounder that may limit the generalizability of these findings to healthcare settings in which take-home dosing is routine. Nevertheless, the consistency observed across multiple thematic domains indicates that the experiential impacts of LAIB are clinically significant and warrant further investigation in larger and more diverse samples.

Patients shared practical suggestions for introducing LAIB in clinical settings. They emphasised delivering thorough and straightforward information, evaluating each person’s preparedness, and highlighting LAIB as a possible way for patients to reclaim independence (refer to Supplementary Table S3).

## 5. Conclusions

In conclusion, integrating narrative medicine with structured thematic analysis showed that LAIB substantially reshapes the lived experience of ongoing opioid agonist treatment. Although sublingual buprenorphine plays a critical role in clinical stabilisation, its daily administration has been perceived as constraining individual autonomy, emotional health, and social reintegration. LAIB, by contrast, was linked to increased autonomy, decreased stigma, enhanced emotional stability, more consistent symptom management, and stronger therapeutic alliances. These results emphasise the significance of evaluating both pharmacological effectiveness and patients’ lived experiences and perspectives to promote care pathways that are personalised and focused on recovery.

## Figures and Tables

**Table 1:** Early life experiences and youth context.

Theme	Description	Patients (n)	Statements
Early exposure to problematic social environments	Growing up in environments where drug use was present.	6	‘The people I spent time with were already experimenting with substances.’ ‘In my area, drugs were easy to find; it was part of the environment.’
School difficulties and early disengagement	School dropout, poor performance, lack of interest, or early behavioural issues.	7	‘School didn’t feel like a place where I belonged.’ ‘I left school early because I couldn’t stay focused or motivated.’
Emotional distress in youth	Feelings of sadness, anxiety, inadequacy, or early emotional fragility.	8	‘Even as a teenager, I felt emotionally overwhelmed without knowing how to cope.’ ‘I often felt uneasy and tense inside, even when nothing was happening.’
Loneliness or social marginality	Feeling different, excluded, or isolated.	5	‘I always felt a bit different from the people around me.’ ‘I didn’t have many meaningful friendships growing up.’
Early responsibilities or premature adulthood	Needing to work early, taking care of family needs, or confronting difficulties too soon.	3	‘I started working young because my family needed help.’ ‘I had to manage many things alone from an early age.’

The statements presented for each thematic area serve as illustrative examples and originate from single interviews. Two quotations per theme were selected to represent the range of narratives.

**Table 2:** Initiation of substance use.

Theme	Description	Patients (n)	Statements
Curiosity or experimentation during adolescence	Substance use began as exploration, imitation of peers, or youthful experimentation.	6	‘At first I tried it out of curiosity, without understanding the risks.’ ‘I experimented because others my age were doing the same.’
Substance use triggered by peer group influence	Initial use occurred within a social group already involved with substances.	9	‘I started because everyone around me was already using.’ ‘Being with that group made it feel normal to try.’
Emotional or psychological triggers	Substances used to cope with anxiety, sadness, trauma, or emotional pain	8	‘Substances made me feel calmer when I couldn’t manage my emotions.’ ‘I used to escape from situations I felt unable to handle.’
Self-medication for discomfort	Substances were used to manage physical symptoms or internal discomfort.	7	‘At the beginning, it helped me feel balanced enough to get through the day.’ ‘I used the substance because it eased a sense of inner discomfort I couldn’t describe.’
Environmental exposure in early adulthood	First use occurred in contexts such as nightlife, work environments, or street settings	7	‘Where I was working, drugs were always around.’ ‘I came across it in the street, and it slowly became part of my routine.’
Escalation from ‘lighter’ substances to opioids	Initial use of non-opioid substances later progressed to heroin or other opioids.	6	‘It started with milder substances and gradually escalated.’ ‘What began as occasional use became something stronger over time.’

The statements presented for each thematic area serve as illustrative examples and originate from single interviews. Two quotations per theme were selected to represent the range of narratives.

**Table 3:** Consequences of substance abuse.

Theme	Description	Patients (n)	Statements
Physical deterioration and health issues	Physical decline, withdrawal-related suffering, exhaustion, or illness linked to substance use.	11	‘My body couldn’t take it anymore; I felt physically drained most of the time.’ ‘I often felt sick, weak, and unable to function normally.’
Impact on work and daily functioning	Difficulty maintaining employment, instability, loss of routine.	8	‘I couldn’t keep a job because the substance controlled everything I did.’ ‘My days revolved around getting and using the substance; working was impossible.’
Negative effects on family relationships	Tension, loss of trust, emotional distance, or family suffering.	8	‘My family suffered a lot because of my addiction; they didn’t know how to help me.’ ‘My relationships at home fell apart as my use got worse.’
Social isolation and marginalisation	Loss of friendships, distancing from healthy networks, and stigmatisation.	9	‘Little by little, I isolated myself and stopped seeing people who cared about me.’ ‘I ended up alone because my whole life was centred around using.’
Financial strain	Spending money on substances, debt, and loss of economic stability.	6	‘All my money went into drugs; I couldn’t manage even basic expenses.’ ‘I lost financial control completely and couldn’t keep up with bills.’
Legal problems or risky behaviours	Police encounters, theft, unsafe environments, and dangerous situations.	6	‘I got into trouble with the law because of the life surrounding the substance.’ ‘I found myself in risky environments and situations just to get what I needed.’
Loss of personal identity and self-worth	Feeling defeated, ashamed, or disconnected from one’s sense of self.	9	‘I didn’t recognise myself anymore; everything revolved around the drug.’ ‘I felt like I had lost all dignity; the substance dictated who I was.’

The statements presented for each thematic area serve as illustrative examples and originate from single interviews. Two quotations per theme were selected to represent the range of narratives.

**Table 4:** Positive and negative statements regarding traditional therapy were evaluated and classified across eight thematic domains (dependence on therapy; emotional impact and self-perception; determination to change; QoL—money, time, work; craving and withdrawal; treatment adherence/use of opioids; social burden; doctor–patient relationship).

Total	Positive Statements	Negative Statements
Dependence on the daily demands of the treatment regimen	0	10
Emotional impact and self-perception	4	7
Determination to change	2	0
QoL (money, time, work)	7	19
Craving and withdrawal symptoms	2	15
Treatment adherence/Use of opioids	8	8
Social burden	1	7
Doctor–patient relationship	3	4

**Table 5:** Positive and negative statements regarding long-acting subcutaneous buprenorphine were evaluated and classified across the eight thematic domains (dependence on therapy; emotional impact and self-perception; determination to change; QoL—money, time, work; craving and withdrawal; treatment adherence/use of opioids; social burden; doctor–patient relationship).

Total	Positive Statements	Negative Statements
Dependence on the daily demands of the treatment regimen	12	1
Emotional impact and self-perception	25	4
Determination to change	16	0
QoL (money, time, work)	40	2
Craving and withdrawal symptoms	11	3
Treatment adherence/Use of opioids	9	6
Social burden	7	0
Doctor–patient relationship	11	0

## Data Availability

The raw data supporting the conclusions of this article will be made available by the authors on request.

## Supplementary Materials

The following supporting information can be downloaded at: https://www.mdpi.com/article/10.3390/ijerph23030336/s1, Table S1: Introduction and patient engagement in long-acting injectable therapy; Table S2: Psychological support during treatment; Table S3: Patient recommendations for presenting the injectable therapy.
