# Supplementary material for: Exploring the Italian Experience with Long-Acting Buprenorphine Formulations (LAIB) for the Treatment of Opioid Use Disorder: A Series of Narrative Interviews
Source: Int J Environ Res Public Health. 2026 Mar 7;23(3):336. doi: 10.3390/ijerph23030336 (PMC13026937; doi:10.3390/ijerph23030336)
Supplement: Supplementary file 1 [file ijerph-23-00336-s001.zip › Table S1_Engagement.pdf]

Table S1. Introduction and patient engagement in long-acting injectable therapy.

Theme	Description	Patients (n)	Statemets
Therapy proposed by clinician	The injectable option was introduced directly by the doctor or clinical team.	14	*'My doctor explained that this new therapy could be suitable for me, and I trusted their judgment.'* *'The clinicians presented the option and guided me through the transition step by step.'*
Motivation to try the injectable therapy	Desire to improve daily stability, reduce dependence on daily dosing, increase autonomy.	13	*'I accepted immediately because I wanted a treatment that would help me regain stability.'* *'I felt this therapy could finally give me a more structured and balanced life.'*
Initial hesitation or fears	Concerns about coverage, fear of injections, doubts about effectiveness.	9	*'My only worry was whether the injection would last long enough, but that fear disappeared quickly.'* *'I was scared of needles at first, but once I tried it, I felt much better than expected.'*
Clear explanation and reassurance from staff	Clinicians provided information about duration, dosage, and expected effects.	9	*'The staff reassured me and put me in touch with patients already on the injection, which encouraged me to start.'* *'Knowing how it worked reassured me and made the transition easier.'*
Expectation of greater freedom and reduced burden	Patients saw the injectable therapy as an opportunity for increased independence and fewer obligations.	13	*'I thought it would help me live more freely, without the weight of daily medication.'* *'The idea of coming to the service only once a month felt like a real relief.'*
Perceived as a step toward recovery or detoxification	Some patients saw the injection as a pathway to long-term recovery.	9	*'I believed this therapy could help me move toward complete detoxification.'*

The statements presented for each thematic area serve as illustrative examples and originate from single interviews. Two quotations per theme were selected to represent the range of narratives.
