# Supplementary material for: Exploring the Italian Experience with Long-Acting Buprenorphine Formulations (LAIB) for the Treatment of Opioid Use Disorder: A Series of Narrative Interviews
Source: Int J Environ Res Public Health. 2026 Mar 7;23(3):336. doi: 10.3390/ijerph23030336 (PMC13026937; doi:10.3390/ijerph23030336)
Supplement: Supplementary file 1 [file ijerph-23-00336-s001.zip › Table S2_Psycologic support.pdf]

Table S2. Psychological support during treatment.

Theme	Description	Patients (n)	Statements
Access to psychological support during traditional therapy	Patients received psychological counselling while on oral therapy (methadone/buprenorphine).	5	*'During traditional therapy, I also received psychological support, which helped me better understand my difficulties.'* *'Talking with the psychologist helped me stay focused and work on the issues behind my use.'*
Family-involved psychological support	Psychological counselling extended to family members, improving communication and understanding.	3	*'Part of the psychological support included my family, which helped us rebuild some trust.'* *'Counselling sessions involving my relatives helped them understand what I was going through.'*
Absence of psychological support in previous treatment pathways	Some patients reported having no psychological support before switching to injectable therapy.	6	*'I never received psychological help before; it was only medication and nothing more.'* *'No one ever offered counselling in the past; everything focused only on the substance.'*
Perceived usefulness of psychological support	When available, psychological assistance was experienced as valuable and complementary to medical therapy.	5	*'The support I received helped me understand myself better and gave me more tools to face the treatment.'* *'Psychological support made a difference; it helped me deal with the emotional side of recovery.'*

The statements presented for each thematic area serve as illustrative examples and originate from single interviews. Two quotations per theme were selected to represent the range of narratives.
