# Supplementary material for: Exploring the Italian Experience with Long-Acting Buprenorphine Formulations (LAIB) for the Treatment of Opioid Use Disorder: A Series of Narrative Interviews
Source: Int J Environ Res Public Health. 2026 Mar 7;23(3):336. doi: 10.3390/ijerph23030336 (PMC13026937; doi:10.3390/ijerph23030336)
Supplement: Supplementary file 1 [file ijerph-23-00336-s001.zip › Table S3_Recommendations.pdf]

Table S3. Patient recommendations for presenting the injectable therapy.

Theme	Description	Patients (n)	Statements
Encouragement to try the therapy	Patients who openly recommend the injectable option to peers still on traditional therapy.	11	*'I would recommend this treatment to others because it truly made a difference for me.'*<br>*'I would recommend this treatment to others because it truly made a difference for me.'*
Emphasising improved quality of life	Patients highlight benefits such as stability, freedom, and an easier daily routine.	10	*'I tell people that this therapy gives you stability and makes everyday life easier.'*<br>*'I explain that it offers more freedom and less stress compared to daily medication.'*
Framing the therapy as a step toward recovery	Patients present the therapy as supportive in the long-term recovery process.	8	*'I explain that this treatment can help you move forward and detach from the substance.'*<br>*'I see it as a step toward getting better, and I tell others the same.'*
Highlighting practical benefits for work and daily commitments	Patients emphasize organizational advantages: fewer visits, better functioning.	9	*'I tell people that coming only once a month makes work and life much easier.'*<br>*'It's easier to manage your job and responsibilities when you're not tied to daily medication.'*
Sharing personal experience to motivate others	Patients use their own progress as a testimonial to encourage acceptance.	10	*'I tell my story because seeing my improvement helps others consider the therapy.'*<br>*'People trust it more when they hear how much it helped someone they know.'*

The statements presented for each thematic area serve as illustrative examples and originate from single interviews. Two quotations per theme were selected to represent the range of narratives.
